# Hypoxia, Hypoxia-Inducible Factors and Liver Fibrosis

**DOI:** 10.3390/cells10071764

**Published:** 2021-07-13

**Authors:** Beatrice Foglia, Erica Novo, Francesca Protopapa, Marina Maggiora, Claudia Bocca, Stefania Cannito, Maurizio Parola

**Affiliations:** Unit of Experimental Medicine and Clinical Pathology, Department of Clinical and Biological Sciences, University of Torino, 10125 Torino, Italy; beatrice.foglia@unito.it (B.F.); erica.novo@unito.it (E.N.); francesca.protopapa@unito.it (F.P.); marina.maggiora@unito.it (M.M.); claudia.bocca@unito.it (C.B.); stefania.cannito@unito.it (S.C.)

**Keywords:** hypoxia, hypoxia-inducible factors, liver fibrosis, liver fibrogenesis, chronic liver diseases

## Abstract

Liver fibrosis is a potentially reversible pathophysiological event, leading to excess deposition of extracellular matrix (ECM) components and taking place as the net result of liver fibrogenesis, a dynamic and highly integrated process occurring during chronic liver injury of any etiology. Liver fibrogenesis and fibrosis, together with chronic inflammatory response, are primarily involved in the progression of chronic liver diseases (CLD). As is well known, a major role in fibrogenesis and fibrosis is played by activated myofibroblasts (MFs), as well as by macrophages and other hepatic cell populations involved in CLD progression. In the present review, we will focus the attention on the emerging pathogenic role of hypoxia, hypoxia-inducible factors (HIFs) and related mediators in the fibrogenic progression of CLD.

## 1. Fibrogenesis and Fibrosis in Chronic Liver Disease Progression: Introductory Remarks

Chronic liver injury is known to result in the persistent activation of liver fibrogenesis, a critical and dynamic biological process involving a plethora of molecular mechanisms, mediators and interactions/responses of different cell populations, which can eventually lead to liver fibrosis (i.e., the net result of persistent fibrogenesis). In turn, liver fibrosis is now defined as a potentially reversible pathophysiological event characterized by an excess deposition of extracellular matrix (ECM) components [1,2,3,4,5,6]. Liver fibrogenesis and fibrosis are primarily involved in chronic liver disease (CLD) progression, as induced by the major etiological agents or conditions affecting human patients [1,2,3,4,5,6,7,8,9,10]. As summarized in Table 1, chronic liver injury may prevalently affect liver parenchyma or may result in cholangiopathies. Parenchymal injury is typically related to chronic infection by hepatitis B virus (HBV) or hepatitis C virus (HCV), to chronic alcohol consumption or to chronically altered metabolic conditions (e.g., non-alcoholic fatty liver disease (NAFLD)). More rarely chronic parenchymal injury is due to autoimmune injury to hepatocytes or due to genetic causes. Cholangiopathies can be either genetically related or can represent the result of autoimmune attack to cholangiocytes of the biliary tree. The etiology can be relevant in inducing different patterns of fibrosis [1,9], including (i) bridging or post-necrotic fibrosis, mainly due to chronic viral infection or autoimmune hepatitis, resulting in the development of fibrotic septa that literally bridge the portal areas to the central vein areas (porto-central septa) or adjacent portal areas; (ii) perisinusoidal or pericellular fibrosis, observed in the early phases of steatohepatitis, either in NAFLD or ALD, with excess deposition of ECM components being found either in the space of Disse or around groups of hepatocytes; and (iii) biliary-like fibrosis, detected in primary biliary cholangitis (PBC) or primary sclerosing cholangitis (PSC), following an immune-mediated attack on the cholangiocytes, or in other cholangiopathies; this pattern of biliary fibrosis is due to chronic damage to cholangiocytes, and results in an excess deposition of ECM components in the portal areas as well as proliferation of cholangiocytes (the so-called ductular reaction); in time, this leads to expansion of the portal tract and only later to the formation of portal–portal fibrotic septa.

CLD progression is a clinically relevant issue that typically proceeds through a reiterated sequence of chronic hepatic (i.e., hepatocyte) injury and persistent activation of inflammatory response, which, in turn, is critical in sustaining the chronic activation of both fibrogenesis and wound-healing response in the liver. If the etiological agent or condition is not removed, CLD can slowly progress through the development of advanced fibrosis and eventually liver cirrhosis. Liver cirrhosis, in turn, can be conveniently defined as an advanced stage of CLD characterized by a number of parenchymal changes, including the presence of regenerative nodules delimited by fibrotic septa as well as by significant changes in organ vascular architecture. These parenchymal and vascular changes can result in portal hypertension and related complications, including ascites, variceal bleeding, hepatic encephalopathy and hepatorenal syndrome. In the scenario of decompensated cirrhosis, these changes can then result in the development of liver failure, with cirrhotic patients carrying also a significant risk to develop hepatocellular carcinoma (HCC) [1,2,3,4,5,6,7,8,9,10].

In the last two decades extensive experimental, translational and clinical studies on fibrogenic CLD progression have unequivocally outlined a number of critical concepts and issues. The interested reader should refer to existing exhaustive reviews and critical studies (see the references provided hereafter) that deeply analyze the following major points: (i) liver fibrogenesis, as a dynamic process, operates in a scenario that involves interactions (and related responses) between hepatic and extrahepatic cell populations and that this is mediated by a huge number of growth factors, cytokines, chemokines, reactive oxygens species (ROS), adipokines and several other mediators through established pro-fibrogenic signaling pathways [4,5,6,7,8]; (ii) in this scenario, a major role is played by persistent activation of either resident (i.e., Kupffer cells) or peripheral blood-recruited macrophages [11,12,13], as well as by the process of activation and transdifferentiation of liver myofibroblasts (MFs) that originated mainly from hepatic stellate cells (HSC) or portal fibroblasts [1,4,6,7,8,9]; (iii) the process leading to fibrosis and CLD progression also involves other cell populations, including sinusoidal endothelial cells (SEC) or cells of either innate and acquired immunity (including T lymphocytes, natural killer or NK cells, NKT cells and B lymphocytes) as well as hepatic progenitor cells and, in biliary-like fibrosis, also activated/damaged cholangiocytes [1,4,6,7,8,9]; (iv) if the etiological agent or condition is efficiently removed or counteracted, liver fibrosis can regress or, at the early stages, even reverse [2,14]; and (v) the histopathological pattern of ECM deposition and then of fibrosis is often intrinsically related to the specific etiology of the CLD [1,9,10].

In addition to these relevant concepts and issues, pre-clinical and translational studies have also outlined a number of major pro-fibrogenic mechanisms that drive CLD progression, once again discussed in authoritative reviews to which the interested reader is referred. Some of these mechanisms can be considered of general relevance and then common to CLD of different etiology, including the role of (i) oxidative stress and ROS [8,9]; (ii) qualitative and/or quantitative alterations of the ECM [7,14,15,16]; (iii) extracellular vesicles released by damaged or activated cells [17,18,19]; (iv) specific biological processes, such as autophagy and endoplasmic reticulum (ER) stress [20,21,22,23]; (v) genetic variants and epigenetic changes [24,25,26]; (vi) hypoxia, hypoxia-inducible factors and related mediators; and (vii) liver pathological angiogenesis [10,27,28,29]. Other mechanisms are more etiology-dependent and include, for example, (i) the role of lipotoxicity (involved mainly in NAFLD but also in ALD and in HCV-related CLD) [30,31]; and (ii) the role of cholangiocytes in cholangiopathies and in biliary-like fibrosis [32,33,34,35]. All these achievements, originally obtained in pre-clinical and translational studies, represented a critical step forward, forming the basis of novel strategies and approaches for targeted therapies designed to interfere with and inhibit the fibrogenic progression of CLD, which actually have been tested in clinical trials [4,5,6,7,8,9].

In the present review, we will specifically focus our attention on the emerging role of hypoxia, hypoxia-inducible factors (HIFs) and related mediators and events in the fibrogenic progression of CLD. Accordingly, we will not intentionally enter into the field of the role of HIF-1α and HIF-2α in liver carcinogenesis, since this would really require, due to the very relevant amount of data and studies available, a separate exhaustive review.

## 2. Hypoxia and Hypoxia-Inducible Factors: The Strange Case of the Liver and the Cellular “Hypoxic Response”

### 2.1. Hypoxia and the Liver: Critical Issues

The interest in the role of hypoxia in the progression of CLDs has grown exponentially in the last two decades as it was originally stimulated mostly from studies that were investigating the relationships between pathological angiogenesis (i.e., an established hypoxia-related process) and liver fibrogenesis in CLD of different etiology [36,37,38]. These pioneer studies were then rapidly extended to investigate at the molecular and cellular levels the hypoxia-related mechanisms and mediators able to affect CLD progression towards fibrosis, cirrhosis and even the development of hepatocellular carcinoma [8,9,10,27,28,29,39,40,41], with a more recent interest focused on NAFLD progression [39,42].

The growing interest in hypoxia, and shortly thereafter on hypoxia-inducible factors (HIFs), also depends on the knowledge that in physiological conditions the liver has a unique double vascular supply by receiving oxygen-depleted blood from portal vein branches and highly oxygenated blood via the hepatic artery. Along these lines, if the term hypoxia can be conveniently defined as an oxygenation state that is under the norm for a defined tissue or organ, the liver is indeed unique since the double vasculature supply generates a gradient of oxygen partial pressure (pO_2_) that, following the direction of the blood flow, ranges from a pO_2_ of 60–65 mmHg (84–91 μmol/L) in periportal areas to a pO_2_ of 30–35 mmHg (42–49 μmol/L) in the perivenous areas [43]. This gradient of pO_2_ is strictly related to liver zonation, and, of course, in normal conditions a “hypoxic response” does not occur. Under physiological conditions and in the presence of normal endothelium (i.e., lined by fenestrated SEC, a condition greatly facilitating exchanges of metabolites and substrates between blood and hepatocytes), the biochemical and functional properties of hepatocytes substantially differ in relation to liver zonation, with hepatocytes of Zone 1 receiving higher levels of O_2_ and displaying a more oxidative metabolism [43]. The pO_2_ gradient and then O_2_ availability, in addition to affecting several biochemical reactions and mitochondrial energy production, can also modulate nutrient- and energy-sensing pathways as well as other critical issues, including the hyperplasic response to injury and its resolution, DNA and histone methylation status, the unfolded protein response and the promotion of collagen maturation [43], to name just a few relevant ones.

The overall scenario can significantly change in conditions of CLD and a response to hypoxic conditions can be rapidly induced even by moderate and/or local pO_2_ changes that have been described in the early stages of chronic injury. As a matter of fact, detection of hypoxic areas (and of related pathologic angiogenesis) has been reported to become increasingly significant by paralleling the progression to more advanced stages of the disease [39,40,41,42,43]. This is not surprising since the progression of CLD proceeds, independently on the etiology, through a persisting sequence of events, involving chronic hepatic injury, cell damage and death, as well as sustained activation of inflammatory and wound healing responses. All these events progressively and significantly alter the physiological scenario previously described.

### 2.2. Hypoxia-Inducible Factors

**Figure 1:**
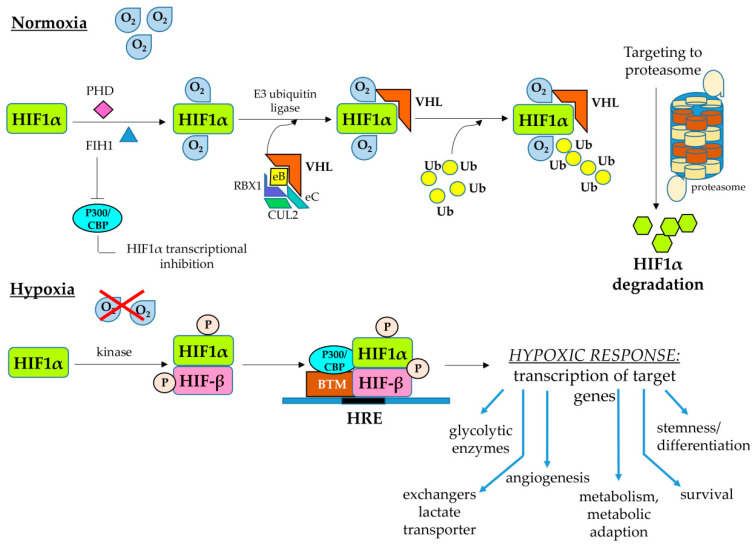
HIFs regulation under normoxic or hypoxic conditions and the hypoxic response. In normoxic conditions, the transcriptional action of heterodimeric hypoxia-inducible factors (HIFs) is prevented by changes introduced into the HIF-α subunit (here HIF-1α is shown) by the action of either prolyl hydroxylases (PHD) or factor inhibiting HIF1 (FIH1). In particular, following the action of PHD, the modified HIF-1α can be recognized by the von Hippel–Lindau (VHL) E3 ubiquitin ligase complex, poly-ubiquitinated (Ub-ubiquitin) and then degraded by the proteasome. Under hypoxic conditions, PHD and FIH are progressively inhibited in an O_2_-dependent manner and the heterodimer HIF-1α/HIF-1β can be phosphorylated and stabilized to then form a transcriptional complex with cAMP-response element binding (CREB)-binding protein (CBP) and histone acetyltransferase p300 (p300/CBP) and bind to hypoxia-responsive elements (HRE) in the promoter or enhancer regions of target genes. More details can be found in the text.

Liver cell populations in the hepatic parenchyma, as for any other organ or tissue, need to receive for their survival a constant supply of O_2_, which is used in primis as the final acceptor of electrons in mitochondrial oxidative phosphorylation. Any significant change from normoxic conditions may affect the respiratory chain functions that, in turn, may cause also increased intracellular generation of ROS by the electron transport chain, an event potentially leading to oxidative stress, cell injury and death. For these reasons, an aerobic organism has developed a number of systemic and cellular mechanisms designed to sense the O_2_ levels and carefully control its homeostasis. In particular, all cells in the organism respond to hypoxic conditions by employing HIFs, which are members of the basic helix–loop–helix Per–Arnt–Sim (bHLH-PAS) family of evolutionary highly conserved heterodimeric transcription factors that regulate the response of thousands of genes [44,45,46,47]. These heterodimers are composed of a hypoxia-inducible and oxygen-sensitive α subunit (HIF-α) and by a β subunit that is constitutively expressed (HIF-β) [44,45]. The O_2_ sensing ability is based on two different dioxygenases that by splitting O_2_ into oxygen atoms result in the hydroxylation of specific prolyl (by prolyl hydroxylases or PHDs isoforms) and asparaginyl residues (by the factor inhibiting HIF1 or FIH1) in the oxygen-sensitive domain of the HIF-α subunit. Under normoxic conditions, the hydroxylation of prolyl residues by PHDs allows the conjugation of modified HIF-α subunits with a E3 ubiquitin ligase complex containing the von Hippel–Lindau (VHL) protein, leading then to HIF-α subunit poly-ubiquitination and its proteasome degradation, subsequently preventing the formation of the heterodimer. The hydroxylation on the asparaginyl residue by FIH1 will instead prevent the binding of the HIFα subunits with the transcriptional co-activator cAMP-response element binding (CREB)-binding protein (CBP) and histone acetyltransferase p300 (p300 HAT), impeding the transcriptional activity of the heterodimer. Even a modest decrease in the pO_2_ can progressively inactivate PHDs whereas FIH1 activity is inhibited in the presence of more severe levels of hypoxia, leading to the formation of the active heterodimeric transcription factor and then the transcriptional complex [44,45,46,47]. Once the transcriptional complex is formed, it can bind to the so-called hypoxia-responsive elements (HRE), containing the core sequence (RCGTG), and present in the promoter or enhancer sequences of several genes. The binding of HIFs to HRE then switch on the cellular hypoxic response (Figure 1), which is a multifaceted one and involves increased transcription of thousands of genes, including, at least, the following: (i) genes involved in the switch towards anaerobic glycolysis (i.e., genes encoding for key enzymes of the glycolytic pathways and glucose transporters); (ii) genes encoding for ion exchangers, lactate transporters or other enzymes or transporters designed to control intracellular pH and avoid acidification; (iii) genes involved in metabolism and metabolic adaptation; (iv) genes involved in the angiogenic and vasodilation responses; (v) genes involved in proliferation and/or survival; (vi) genes involved in inflammatory responses; and (vii) genes involved in stemness and differentiation (Figure 1).

At present, three different HIFα subunits have been described, namely, HIF-1α (the first isoform characterized), HIF-2α and HIF-3α; these isoforms are encoded by the HIF1A, HIF2A (also referred to as endothelial PAS domain-containing protein 1 or EPAS1) and HIF3A genes, respectively. All HIFα subunits are members of the Class 1 group of bHLH-PAS proteins and can dimerize with subunits belonging to the Class 2 bHLH-PAS proteins, defined as HIF-1β and aryl hydrocarbon receptor nuclear translocator 2 (ARNT2), respectively, in order to act as heterodimeric transcription factors [48]. HIF-1β is the most abundant dimerization partner but ARNT2 is highly expressed, particularly in the central nervous system and kidney, being able to form HIF complexes that are believed to contribute to neural and neuroendocrine responses to hypoxia [49]. The heterodimeric complexes of HIF-1α, HIF-2α and HIF-3α with HIF-1β/ARNT are named HIF1, HIF2 and HIF3, respectively, with HIF-2α being able to complex with ARNT2, mainly in the central nervous system and kidney.

Relevant to what we will discuss later, on the role of HIFs in CLDs and liver fibrosis, there are a number of critical peculiarities of these transcription factors, with most of the available knowledge being related to HIF-1α and HIF-2α, and then to HIF1 and HIF2. First, HIF-1α is ubiquitously expressed in all cells and tissues whereas HIF2α is more selectively expressed in endothelial cells as well as other cells, such as hepatocytes, type II pneumocytes, glial cells, myocardial cells, duodenal and pancreatic interstitial cells or non-tubular kidney cells [44,45,46,47]. Second, although both HIF1 and HIF2 (and then HIF-1α and HIF-2α) may both respond to hypoxia, their transcriptional gene targets, oxygen dependence and kinetics of activation differ substantially. Data from the literature indicate, for example, that HIF-1α is strongly induced by severe hypoxia and its action last usually for the first 24 h, whereas HIF-2α is induced by more moderate conditions of hypoxia and last for a longer time [50,51,52,53]. Even more relevant, HIF1α and HIF2α, and then HIF1 and HIF2, can upregulate the transcription of common as well as of distinct target genes. As nicely synthetized some years ago by Lee and Simon [53], we can subdivide the target genes in three main categories:

(a) Genes responding to both HIF1α and HIF2α, in which one can include the genes encoding for (i) VEGF-A; (ii) glucose transporter 1 (GLUT1); (iii) carbonic anhydrase IX and XII; (iv) the inflammatory response-related ones encoding for chemokine receptor CXCR4, the chemokine CXCL12 or IL-1β; (v) the anti-apoptotic protein Bcl2; and (vi) the transcription factor Twist involved in epithelial–mesenchymal transition (EMT).
(b) Genes responding to HIF1α, including those encoding for (i) key enzymes of the glycolytic pathway; (ii) factors involved in autophagy, such as BCL2/adenovirus E1B 19 kDa protein-interacting protein 3 or BNIP3 and the related ligand BNIP3L; (iii) ECM remodeling enzymes, such as lisyl oxidase 2 or LOX2; (iv) the inhibitor of mammalian-target of rapamycin (mTOR) signaling pathway REDD1 (regulated in development and DNA damage responses 1); and (v) inducible NO synthase (iNOs).
(c) Genes responding to HIF2α, including those encoding for (i) factors involved in cell cycle and proliferation or stemness, such as cyclin D1, transforming growth factor-α (TGFα) and Octamer 4; (ii) ECM remodeling enzymes, such as matrix metalloprotease 13 (MMP13); (iii) antioxidant enzymes, such as superoxide dismutase 2 (SOD2); (iv) the delta-like ligand 4 (DLL4) involved in Notch signaling; and (v) the marker for M2 macrophage polarization arginase 1.

As will be emphasized later in this review, HIF-1α and HIF-2α, and then HIF1, HIF2 and related mediators, play a major role in CLD progression and liver fibrosis.

At present, much less is known about HIF-3α (and then HIF3), which differs from the other α subunits given the lack of a COOH-terminal transactivation domain (C-TAD) and for the presence of a unique leucine zipper (LZIP) domain in the COOH-terminal region [54]. This lack of knowledge is intrinsically related to the existence of multiple HIF-3α variants and indeed at least ten different HIF-3α transcripts have been reported in humans, although it has estimated that there may be as many as nineteen, due to the use of different promoters and transcription initiation sites as well as of alternative splicing [54]. The complexity is further emphasized by the fact that HIF-3α variants are expressed in different tissues or organs, at different developmental stages and are differentially regulated by hypoxia and other factors. The biological action is even more complex since these variants can display different and even opposite functions, including the following: (i) Full-length HIF-3α, particularly human HIF-3α1, functions as an oxygen-regulated transcription activator, being induced by hypoxia at both the transcription and protein stability levels. Moreover, full-length HIF-3α activates a transcriptional program that is partially overlapping with the one elicited by HIF-1α. (ii) Some HIF-3α variants may operate by inhibiting the HIF-1α and HIF-2α action simply by competing for binding with the common HIF-β subunit. (iii) Human HIF-3α7 and -3α8 variants apparently do not display any transcriptional activity. (iv) Human HIF-3α4 is a truncated variant reported to act as a dominant-negative regulator of HIF-1/2α [54]. However, at present, no significant data have been directly or indirectly reported in relation to the role of HIF-3α and HIF3 in CLDs.

### 2.3. HIFs and the Cellular Response to Hypoxic Conditions: A Focus on Maintenance of Redox Ho-Meostasis

The scenario of adaptive responses to hypoxic conditions in either physiological or pathological conditions is extremely complex, and the major aspects of the HIF-mediated cell responses were already briefly mentioned in the previous section. The typical adaptive hypoxic response include the transcription of genes favoring the shift towards an anaerobic glycolytic pathway and of those preventing intracellular acidification, as well as the upregulation of genes involved in the control of apoptosis, in the modulation of the cell cycle and cell proliferation or in the modulation of biological processes, such as migration and invasiveness as well as of epithelial to mesenchymal transition. The interested reader may find more details on these and other aspects of the cell response to hypoxia in authoritative and exhaustive reviews [43,44,45,46,47,51,52,55,56]. In this and the following sections, the focus will be on the two major aspects that are relevant for fibrogenic CLD progression: the sometimes neglected and still under-investigated role of HIFs in the maintenance of redox homeostasis and the overall hyper-investigated role of HIFs, particularly HIF1, in pathological angiogenesis.

As it is well known, oxidative phosphorylation allows mammalian cells to generate ATP in those amounts required for the development and maintenance of multicellular organisms. However, under hypoxic conditions, such as those that progressively develop during the course of a CLD, mitochondrial electron transport becomes less efficient, leading to increased generation of superoxide anions and then of other ROS that may potentially lead to cell injury and death. HIFs, under hypoxic conditions, can significantly contribute to redox homeostasis by activating the transcription of target genes encoding for proteins serving to down-modulate mitochondrial generation of ROS (i.e., switching cells from oxidative to glycolytic metabolism) or to increase production of antioxidants and ROS scavengers [57]. This is a rather critical issue since, as previously mentioned in the introduction, oxidative stress (and then generation of ROS and other related reactive mediators) is considered as a major pro-fibrogenic mechanism that drives CLD progression, whatever the etiology [8,9,58,59,60]. There are a number of key metabolic crossroads in which HIFs, particularly HIF1, have been suggested to be critical in the maintenance of redox homeostasis [57]. Some of these issues (i.e., those related to metabolism) will be recalled and further analyzed later in this review because of their relevance for CLD progression, whereas others are still poorly or even not investigated in that connection. The major issues can be summarized as follows:

(a) Under hypoxic conditions, HIF1 can modulate the equilibrium between oxidative phosphorylation and glycolytic metabolism, and then ROS generation, mainly by regulating the expression of the *LDHA* and *PDK1* genes encoding for lactate dehydrogenase A (i.e., the enzyme converting pyruvate to lactate) and pyruvate dehydrogenase kinase 1 (i.e., the enzyme which inactivates pyruvate dehydrogenase preventing conversion of pyruvate into acetyl-CoA). Accordingly, deletion of HIF1α in genetically manipulated cells prevented the hypoxia-related switch from oxidative to glycolytic metabolism and maintained high levels of ATP. However, HIF1α-deleted cells can die for an excess of intracellular ROS generation, also revealing the critical role of mitochondrial autophagy for the maintenance of redox homeostasis and the survival of hypoxic cells [61,62].
(b) Another way to modulate ROS generation is by controlling fatty acid oxidation. HIF-1α, and then HIF1, under hypoxic conditions can suppress, through a pathway involving downregulation of C-MYC and then of peroxisome proliferator-activated receptor-gamma (PPAR-gamma) coactivator-1 beta (PGC-1β) expression, the transcription of the medium-chain and long-chain acyl-CoA dehydrogenases (MCAD and LCAD) genes, the enzymes that catalyze the first step of fatty acid oxidation in the mitochondria, subsequently decreasing fatty acid oxidation and mitochondrial ROS generation [57,63].
(c) HIF1 can downregulate mitochondrial ROS generation by stimulating mitochondrial-selective autophagy (or mitophagy), through upregulation of BNIP3 in normal cells or of BNIP3L in tumor cells [61,64]. Conceivably, one can assume that under hypoxic conditions the reduction in the cellular content of mitochondria can suppress both glucose and fatty acids oxidation, resulting in a decreased mitochondrial generation of ROS. What is interesting here is that actual knowledge suggests that liver parenchyma is highly dependent on autophagy for maintaining its normal function and to prevent the development of disease states. Indeed, several laboratories have shown that alterations in autophagy represent a mechanism underlying hepatic diseases of different etiology, both acute or chronic, and even hepatocellular carcinoma development [65,66]. In addition, it has been also shown that autophagy can efficiently fuel fibrogenesis [22,66,67,68,69,70] but, paradoxically, few experimental studies have investigated the possible link between HIF, autophagy and CLDs [71,72], and no one was directly related to fibrogenesis.
(d) Under hypoxic conditions HIFs could reduce mitochondrial ROS generation through two additional mechanisms: (i) HIF1 may act by activating the transcription of the gene encoding for NADH dehydrogenase [ubiquinone] 1α subcomplex, 4-like 2 (NDUFA4L2), resulting in a decrease in flux through the electron transport chain and subsequently in ROS generation [73]; (ii) induction of miR-210 expression, which, in turn, represses *ISCU*, a gene encoding the iron–sulfur cluster assembly factors that are required for the biogenesis of iron–sulfur-dependent enzyme complexes, which are critical for electron transport, such as complex I, complex III and aconitase [74].
(e) HIFs, under hypoxic conditions, can also contribute to mitochondrial redox homeostasis by regulating the genes encoding for enzymes that are involved in the generation of NADPH and glutathione [57].

As anticipated, few of the mentioned relationships between hypoxia, HIFs and redox homeostasis have been investigated in relation to conditions of CLD progression, but, in this connection, one should emphasize a final major issue to complete the overall scenario: HIFs have been reported to act as transcription factors in an oxygen-independent way. Along these lines there are at least two mechanisms that deserves attention and are intimately related to the fact that during CLD progression ROS are hugely produced, even in normoxic conditions. Indeed, ROS can be released either by damaged/injured hepatocytes or as a consequence of the interaction of several ligands (growth factors, cytokines, adipokines, etc.) with their receptors, leading to activation of ROS-generating NADPH-oxidase [8,9,10,40,41]. Of interest, ROS have been reported to interfere with HIF transcriptional activity by inhibiting the activity of PHDs, allowing the HIFs to stabilize and translocate into nuclei to operate their action. Moreover, ROS are known to activate the mitogen-activated protein kinase (MAPK) signaling pathways that are believed to lead to phosphorylation and stabilization of HIFs, eventually favoring their transcriptional activity.

## 3. Hypoxia, HIFs and Liver Pathological Angiogenesis

As already mentioned, pathological neo-angiogenesis has been detected, irrespective of etiology, in all major conditions of CLDs, and found to parallel and driving fibrogenic progression and HCC development [8,9,10,36,37,38,39,40]. Moreover, pathological angiogenesis and the resulting vascular reorganization in chronically injured livers have an obvious role in the genesis of portal hypertension and related complications.

Still, from the first pioneer studies, it was clear that hypoxic areas were progressively increasing in liver parenchyma from early injury to more advanced stages and eventually cirrhosis [36]. This intrinsically suggests that CLD progression by itself is likely to significantly contribute to self-perpetuation of tissue hypoxia. Indeed, the latter was interpreted as the consequence of oxygen diffusion impairment in chronically injured livers due to the sum of events such as formation of regenerative nodules, deposition of fibrotic septa, vascular remodeling and progressive capillarization of liver sinusoids. Moreover, it has been suggested that hepatic hypoxia in ongoing CLD, through the action of HIFs, may be in part responsible for the upregulation of factors or mediators able to chronically activate a wound-healing response. Accordingly, persistent hypoxia and pathological angiogenesis may act in a synergic way to dysregulate normal tissue repair and promoting liver fibrogenesis [29,36,37,38,39,42]. Unfortunately, the very encouraging results obtained by testing different experimental antiangiogenic strategies in pre-clinical studies, showing at least partial if not quite significant prevention of fibrogenic progression, were not reproduced in clinical trials (extensively reviewed in References [8,9,10,27,28,29,36,37,38,39,40,41,42]).

In any case, basic and pre-clinical studies have outlined a number of hypoxia- and HIFs-related concepts that still remain of interest. As will be detailed later, in the scenario of persistent tissue hypoxia, HIFs can induce an inflammatory, angiogenic and fibrogenic response by modulating the interrelationships between different populations of liver cells (all able to respond to hypoxic conditions) and their release of pro-angiogenic mediators [27,28,29,36,37,38,39,40,41,42].

Several laboratories have provided data indicating that activated, MF-like HSC (or HSC/MFs) can be considered as reasonable pro-angiogenic cells [26,27,28,29,30,31,32,33,34,35,36,37,38,39,42]. We and others showed that HSC/MFs and, more generally, liver MFs can efficiently respond to hypoxic conditions through a HIF1α-dependent (then HIF1) upregulation of critical pro-angiogenic mediators such as VEGF-A and Angiopoietin-1, as well as of their cognate receptors VEGFR2 and Tie2. In addition, liver MFs respond to hypoxia also by increasing the expression of the CCR1 and CCR5 chemokine receptors as well as of interleukin-13 receptor-α1, prolyl-4-hydroxylase-α2 and placental growth factor [75,76,77]. An increased expression of VEGF-A by activated HSC or HSC/MFs has been reported to occur also in response to leptin and PDGF-BB (i.e., two pro-angiogenic in vivo mediators) through a signaling pathway involving, for both mediators, activation of the mTOR pathway and ROS generation through NADPH oxidase (NOX) [78]. This is another aspect that reinforces the previously mentioned concept: HIFs can act as transcription factors in an oxygen-independent way.

Moreover, liver MFs not only produce VEGF-A in response to hypoxia or other pro-angiogenic mediators, but even more interesting, also respond to VEGF-A by enhancing two typical phenotypic responses, including proliferation and synthesis of ECM components [29,39,77]. In addition, VEGF-A also elicit a characteristic and ROS-dependent chemotactic effect on HSC/MFs, which is strictly related to the activation of NOX following the interaction of VEGF-A with its cognate receptor VEGFR2. This is a redox-related feature observed also for two other well established chemoattractant peptides for HSC/MFs such as PDGF-BB and CCL2 [79,80]. As a matter of fact, morphological analysis performed on specimens from either human or murine conditions of CLD has revealed that liver MFs are always in close contact with SEC, with neoangiogenic vessels being included in developing septa [75], leading to the proposal that fibrogenesis and angiogenesis may be driven or modulated by both MFs and hypoxia, at least in an early phase in which neoangiogenic vessels are included in developing septa [29,75].

In this connection, although the close contact between HSC and SEC can be appreciated also under physiological conditions, it has been proposed that liver SEC may be critical in organ homeostasis by preventing activation of Kupffer cells and HSC, subsequently also preventing inflammatory and fibrogenic responses [81,82]. Since SEC can regulate intrahepatic vascular resistance and portal pressure during ongoing chronic liver injury, they are believed to become dysfunctional, undergoing capillarization by losing either their typical fenestration or their ability to regulate HSC quiescence. It has been proposed that dysfunctional SEC may release pro-inflammatory and pro-fibrogenic mediators as well as VEGF-A, strongly affecting HSC behavior and overall concurring with activated HSC in sustaining pathologic angiogenesis [29,81,82].

## 4. HIF1α and HIF2α: Two Distinct Critical Players in Fibrogenic CLD Progression

In a previous section (see Section 2.2), we have already introduced a number of concepts that are critical in analyzing the contribution of different HIFs to fibrogenic CLD progression, including two major issues: (i) HIF-1α (then HIF1) is ubiquitously expressed, practically in all tissues, whereas HIF-2α (then HIF2) is more selectively expressed in defined cell types, including hepatocytes; and (ii) both HIF-α subunits respond to hypoxia but their transcriptional gene targets, oxygen dependence and kinetics of activation differ substantially. These two issues are becoming so critical in the field that in this review we will analyze the role of HIF-1α and HIF-2α separately.

**Figure 2:**
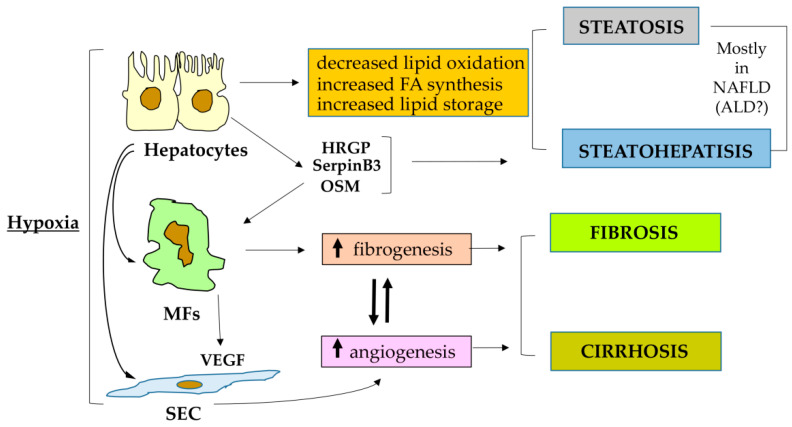
Hypoxia and HIF-induced events in liver fibrogenesis and CLD progression, as mainly focused on progressive NAFLD. Hypoxic conditions can upregulate the transcription of the HIF-dependent target genes potentially in any hepatic cell population. According to the focus on progressive NAFLD (and ALD) and steatohepatitis, hepatocytes react by down modulation of the enzyme and factors involved in lipid oxidation but, at the same time, by favoring an increase in lipid storage and fatty acid (FA) synthesis. Hypoxic hepatocytes also upregulate a number of peptides acting as pro-inflammatory as well as pro-fibrogenic mediators (i.e., through their action on either macrophages or myofibroblasts (MFs), including SerpinB3, histidine-rich glycoprotein (HRGP) and oncostatin M (OSM). Some of these mediators, in addition to vascular endothelial growth factor A (VEGF-A) released by all cells exposed to hypoxia, may also act as pro-angiogenic mediators, with chronic inflammatory responses and persistent activation of fibrogenesis and angiogenesis having a major role in driving CLD progression towards more advanced fibrosis and cirrhosis. More details can be found in the text.

There is today a general consensus in the scientific community of basic and translational hepatologists concerning the relevance of hypoxia and HIFs in CLD progression (Figure 2). However, only in recent years the use of genetically manipulated mice, the improvement of analytical techniques and the continuous effort to translate pre-clinical data into clinical conditions (and vice versa) has offered a consistent advance in knowledge. One should note from the beginning that in the last decade most of the studies, also due to the undisputed efficacy of direct antiviral agents in the therapy of chronic viral infections, have been focused on fibrogenic progression of NAFLD, which is envisaged to emerge as the most relevant CLD worldwide in the next future. Therefore, in the following sections we will report the most relevant data of historical relevance, focusing mostly on data that have been provided by NAFLD- and non-alcoholic steatohepatitis (NASH)-oriented preclinical and translational studies.

### 4.1. HIF-1α and HIF1 in Fibrogenic CLD Progression: Of Biliary-Like Fibrosis and Activated Hepatic Stellate Cells

Starting from pioneer studies that outlined the intimate connections between hypoxia, angiogenesis and fibrogenesis [8,9,10,36,37,38,39,40], several laboratories specifically investigated the involvement of HIF-1α and HIF1 in CLD progression and we will hereafter mention only the most relevant and unequivocal ones.

This section will start by describing a number of studies that proposed a role for HIF-1α in the progression of biliary-like fibrosis in conditions of cholestasis. The first experimental study that proposed the involvement of HIF-1α took advantage from genetically manipulated mice carrying hepatocyte-specific conditional deletion of this subunit (using Cre/lox technology). These mice were submitted to the surgical protocol of bile duct ligation (BDL) designed to induce in rodents a biliary-like pattern of fibrosis somewhat resembling secondary biliary cirrhosis in humans. In these experiments, hypoxia was restricted to liver parenchyma, and HIF-1α nuclear staining was observed only in hepatocytes of the control (i.e., floxed) mice. Following BDL, mice carrying hepatocyte-specific HIF-1α conditional deletion showed a significant reduction in liver fibrosis versus control mice, as evaluated in terms of the transcript and protein levels of type I collagen and α-smooth muscle actin (αSMA). A reduction in fibrosis in HIF-1α-deficient mice was paralleled by decreased expression of platelet-derived growth factor (PDGF)-A and PDGF-B [83]. The same research group contributed other studies that supported this role of HIF1α. The first study showed that mouse HSC exposed to hypoxia in culture upregulated several genes in a HIF-1α-dependent manner, including those encoding for chemokine receptor (CCR) 1, CCR5, macrophage migration inhibitory factor, interleukin-13 receptor α1 and prolyl-4-hydroxylase α2, as well as, partially, VEGF-A [84]. In another study, the same group employed mice genetically manipulated to have deletion of HIF1α or HIF-1β in myeloid cells. Following the BDL protocol, mice carrying the HIF-1α or HIF-1β deletion were again characterized by a reduction in collagen type I and αSMA. The authors also reported that the deficiency in the two subunits did not affect liver injury or inflammation after BDL. However, the deletion resulted in a significant reduction in the PDGF-B transcript and protein levels, proposed as a major HIF-dependent mediator in sustaining fibrogenesis. Indeed, PDGF-BB is considered as the most potent mitogenic and chemoattractant stimulus for HSC/MFs and, more generally, liver MFs. Of interest, this study also reported a nuclear HIF-1α-positive stain in macrophages, hepatocytes and fibroblasts in liver specimens obtained from patients with primary biliary cholangitis (PBC) and primary sclerosing cholangitis (PSC) [78,85]. The role of HIF-1α in cholestatic conditions was reinforced by a pre-clinical study that employed the HIF-1α inhibitor 3-(5-hydroxymethyl-2-furyl)-1-benzylindazole (YC-1) in the BDL model. The use of this inhibitor resulted in a significant decrease of HIF1α as well as of liver fibrosis and angiogenesis [86]. Moreover, the YC-1 inhibitor operated by downregulating a pathway involving suppressor of cytokine signaling-1 (SOCS1) and -3 (SOCS3), nuclear factor-κB (NF-κB) activation and phosphorylation of signal transducer and activator of transcription (STAT)-3 [86].

Apart from studies involving HIF1α in fibrogenic progression related to chronic cholestasis, other studies have recently provided evidence that this subunit and then HIF1 can contribute to liver fibrogenesis by modulating the response of different hepatic cell populations. A first example is represented by a recent study showing that upregulation of CCL12 by hypoxia was partially prevented in hepatocytes from HIF-1α-deficient mice and completely prevented in hepatocytes from HIF-1β-deficient hepatocytes. These data suggested that upregulation of CCL12 by hypoxia was perhaps dependent on both HIF-1α and HIF-2α [87].

A number of significant studies, in addition to those already mentioned, have evaluated through the years defined aspects of HSC activation in relation to HIF-1α. The most interesting issues emerged from these studies can be summarized as follows: (i) By employing the rat cell line T6 of activated HSC, it was found that HIF-1α deletion in these cells inhibited activation as well as transcription of IL-6, transforming growth factor β (TGF-β) and connective tissue growth factor (CTGF) and secretion of collagen I induced by hypoxia; moreover, inhibition of MAPK phosphorylation enhanced HIF-1α ubiquitination and degradation, preventing HIF-1α nuclear translocation, suggesting that HIF-1α and MAPK participate and/or cooperate in HSC activation upon hypoxia [88]. (ii) Activated HSC seems able to orchestrate the clearance of necrotic cells during the course of experimental chronic liver injury in a HIF-1α-dependent manner by modulating macrophage polarization towards the M1 phenotype [89]. (iii) A study performed on the human hepatic LX2 cell line has suggested that HIF-1α can regulate the activation of HSC by modulating autophagy [90], and a subsequent study suggested that HIF-1α-dependent upregulation of Bnip3 was the mechanism that likely promoted autophagy and activation of HSCs [91]. (iv) Downregulation of miR-125a-5p, a miRNA known to be upregulated in fibrotic murine livers, prevented the activation and proliferation of HSCs likely by downregulating FIH1, a negative modulator of HIF-1α [92]. (v) A study performed on the rat T6 cell line showed that HIF-1α knockdown proliferation suppressed collagen 1A1 expression, decreased collagen 3A1 secretion and attenuated Rho-associated protein kinase 1 (ROCK1) expression, suggesting that the interplay between HIF1-α and ROCK1 may represent a critical factor in regulating HSC activation under hypoxic conditions [93]. (vi) HIF-1α-dependent upregulation of lncRNA-H19, known to be significantly overexpressed in fibrotic liver, can regulate lipid droplet metabolism through the AMPKα pathway in hepatic stellate cells, concurring with the modulation of HSC activation [94]. (vii) Exposure of LX2 cells to extracellular vesicles (EVs) released by fat-laden hepatocytes undergoing chemical (i.e., CoCl_2_-induced) hypoxia, a procedure stabilizing HIF-1α and then HIF1 action increased the expression of pro-fibrogenic genes [95]. (viii) N-Myc downstream-regulated gene 2 (NDRG2), a potential regulator of fibrosis and a downstream target gene of hypoxia-inducible factor 1 (HIF-1), has been reported to regulate in LX2 cells the expression of TGF-β1 via the NF-κB pathway [96].

Concerning liver macrophages, unfortunately at present few studies have specifically analyzed the role of HIFs in relation to the modulation of their function as well as of the function of other cells of innate immunity, with most of the data related to either experimental or human liver carcinogenesis [38,40,97]. A few selected studies and issues deserved to be mentioned here in relation to CLD progression, including the following: (i) Genetic inactivation in myeloid cells of the von Hippel–Lindau protein (VHL), a negative regulator of hypoxia-inducible factors (HIF), led to increased VEGF expression, accelerated matrix degradation and a reduced number of MFs, with increased expression of MMP-13 in scar-associated macrophages [98]. (ii) Experiments performed on mice genetically manipulated to have a depletion of the aryl hydrocarbon receptor nuclear translocator (ARNT or HIF-1β) in myeloid cells developed steatohepatitis when fed on a high-fat diet; this was accompanied by macrophage infiltration and expression of both M1 and M2 markers, suggesting that myeloid ARNT may have a role in the progression from NAFLD to NASH [99].

### 4.2. HIF-1α and HIF1, Metabolic Diseases and Fibrogenic Progression in ALD or NAFLD

In recent years, the role of HIFs has been deeply investigated in metabolic diseases and of course this is pertinent to the present review, particularly in relation to NAFLD, which is usually diagnosed in obese and/or type II diabetes patients and considered as the hepatic manifestation of metabolic syndrome. As nicely outlined in a quite recent authoritative review [42], the focus on HIFs and metabolic diseases has led to the acquisition of a number of major critical concepts that have also started to discriminate the sometimes alternative roles of HIF-1α and HIF-2α. The following general issues could be considered as critical for NAFLD/NASH progression: (i) Obesity by itself has been reported to be able to trigger hypoxia in adipose tissue and the small intestine; in turn, the raising of hypoxic conditions in these tissues can result, through the action of HIF-1α and HIF-2α, in major and adverse metabolic effects, which include the development of insulin resistance and NAFLD. (ii) In particular, HIF-1α induction in adipocytes is critical in upregulating the expression of inflammatory mediators and in downregulating adiponectin expression, and this scenario can offer a major contribution to the development of insulin resistance and NAFLD, likely by involving the suppressor of cytokine signaling 3 (SOCS3)–signal transducer and activator of transcription 3 (STAT3) axis. (iii) On the other hand, HIF2α activation in the small intestine is believed to result in a significant increase in serum and small intestinal levels of ceramides, a critical event believed to potentiate obesity-associated metabolic diseases. (iv) Experimental and translational studies suggest that either genetic manipulation or pharmacological inhibition of HIF1α and HIF2α in both adipose tissue and small intestine can ameliorate obesity-associated metabolic diseases ([42], and references therein). (v) Hypoxia and HIF signaling might have a vital role in the function of pancreatic β-cells, since disruption of HIF signaling can result in impairment of glucose tolerance, glucose-stimulated insulin release and changes in the gene expression pattern; however, it is unclear which HIFα subunit could be responsible for these effects since both the specific deletion of HIF-1α and HIF-2α independently decreased insulin secretion [42,100,101].

Apart from the general issues just mentioned, the involvement of HIF-1α, particularly in the progression of obesity and type 2 diabetes-associated NAFLD, has been traditionally related to its role (and then of HIF1) in regulating the expression of the genes involved in glucose and lipid metabolism. In this connection, HIF-1α is believed to promote glucose consumption and then the glycolytic pathway whereas HIF2α involvement (as detailed in the next section) has been mostly associated with the regulation of lipid metabolism and storage [44,45,52,53].

Historically, the involvement of hypoxia has been originally suggested by two morphological studies that detected the presence of hepatic hypoxia in Zone 3 (i.e., around the centrilobular vein), which then developed in parallel with fatty liver in murine models of NASH [102] or, as previously reported, of chronic ethanol administration [103]. The involvement of HIFs was then next investigated by different laboratories in preclinical studies that employed genetically manipulated mice.

The involvement of HIFs in NAFLD progression was first unequivocally proposed by a very elegant and mechanistic pioneer study that employed mice genetically manipulated in order to carry a hepatocyte conditional deletion for VHL protein. VHL deletion in hepatocytes, which is preventing the proteasome degradation of HIFα subunits, resulted in the overexpression of both HIF1α and HIF2α and then in the rapid development of an impressive fatty liver associated with an impairment of fatty acid oxidation [104]. Almost homologous results were obtained in other studies employing mice carrying hepatocyte conditional deletion of genes encoding for either PHD2 or PHD3 [105,106]. It should be noted, however, that fatty liver developed in all these transgenic mice in the absence of any induction of liver injury or metabolic alteration, and substantially so in an oxygen-independent way.

To overcome the limitation of a “pure” genetic model of steatosis (i.e., obtained in the absence of chronic liver injury), the first mechanistic study was performed to investigate the role of HIFs in alcohol-mediated injury [107]. The authors, after having described a significant increase in the expression of HIF-1α in alcohol-induced injury, used mice genetically manipulated to either overexpress HIF-1α in hepatocytes or to carry its hepatocyte-specific deletion. Overexpression of HIF-1α resulted in a net increase in liver triglyceride content vs. control littermates and, consistently, HIF-1α hepatocyte deletion prevented both fatty liver and alanine-aminotransferase (ALT) release. In the same study, the authors also provided in vitro data suggesting that CCL2 was able to induce lipid accumulation in the Huh7 cell line and also an increased expression of HIF-1α [107]. However, this study was subsequently challenged by data provided from a different laboratory that employed hepatocyte-specific HIF-1α-null mice subjected to a 6% ethanol-containing liquid diet for 4 weeks. Although these mice developed a severe fatty liver, on the basis of their experimental evidence, the authors reached the opposite conclusion, stating that HIF-1α was rather a protective mediator against ethanol-induced fatty liver [108].

A similar degree of controversy on the role of HIF-1α was reported in three experimental studies that employed genetically manipulated mice fed on a diet able to induce a murine scenario reproducing the major features of human progressive NAFLD. In the first study, mice carrying hepatocyte-specific HIF-1α deletions were fed a high-fat diet up to six months. The data provided were consistent with the hypothesis that HIF-1 expression was responsible for an increase in liver fibrosis, possibly as a consequence of liver hypoxia developed in hepatic steatosis [109]. Results from this first NAFLD-related study were essentially confirmed by a more recent one, still performed on mice carrying hepatocyte-specific HIF-1α deletions and fed a high-fat diet. Once again, the HIF-1α deletion prevented excess deposition of collagen and the authors proposed a role for a novel HIF1a/PTEN/NF-κ Bp65 signaling pathway [110]. These two studies are somewhat conflicting with a third one that was performed in mice fed on a choline-deficient diet (CDD) for 4 weeks, another murine model of progressive NAFLD. In this latter study, the authors provided evidence sustaining a quite different concept: HIF-1α (and then HIF1) was acting rather as a factor able to prevent excessive hepatic lipid accumulation in NAFLD progression; this protecting effect was attributed to the role played by HIF-1α in the regulation of peroxisomal lipid metabolism, likely exerted through the induced expression of lipin1, a key regulator of the PPARα/PGC-1α pathway [111]. A very recent in vitro study offered further evidence indicating a protective role of HIF-1α versus lipid accumulation, which was attributed to its ability to activate the PPAR-α/ANGPTL4 signaling pathway in NAFLD [112].

Overall, the conflicting results about the relevance of HIF-1α in ALD and NAFLD progression should be also analyzed on the basis of the major role that is now attributed to HIF-2α, particularly in relation to the development of liver steatosis in the scenario of NAFLD progression (see Section 4.3).

### 4.3. Oncostatin M as a Profibrogenic Mediator Operating through HIF-1α

In a previous section, it was emphasized that HIF transcriptional activity can be induced by signals or mediators that can operate independently on hypoxia, including ROS and a number of peptide factors that, following interaction with their cognate receptor, act by activating ROS generation through the NADPH-oxidase complex, including PDGF-BB, leptin and VEGF-A. In this light, here we would like to mention the recently emerged pro-fibrogenic role of oncostatin M (OSM) [113,114], a pleiotropic cytokine belonging to the IL-6 family of cytokines that signals through two different heterodimeric receptors formed by gp130 and, alternatively, OSM receptor β (OSMRβ) or leukemia inhibitor factor receptor β (LIFRβ) [115,116].

A role for OSM in CLD was initially suspected on the basis of the fact that this cytokine was involved in liver development regeneration and angiogenesis [117], and that, more generally, it was reported to have a role in the control of lipid metabolism [116] and inflammatory response [118]. However, the real interest in OSM started historically from two studies, one showing that OSM and its receptors were over-expressed in cirrhotic livers [119] and the second showing that OSM upregulated the expression of collagen I and tissue inhibitor of metalloproteinase-1 (TIMP-1) in HSC/MFs [120]. In addition, it was suggested that OSM was also involved in the pathogenesis of steatosis and hepatic insulin resistance [121].

Some years later, the connection between the OSM and HIF-1α pathway was provided by a study demonstrating that OSM was able to upregulate HIF-1α and downstream signaling in hepatocytes and hepatoma cells [122]. Surprisingly, only several years later a mechanistic experimental study, employing OSM knockout mice submitted to the thioacetamide treatment protocol of chronic liver injury, has confirmed the ability of OSM to act as a pro-fibrogenic mediator [113]. Of interest, liver fibrosis was significantly prevented in OSM knockout mice versus related control littermates. Results from this study indicated, in particular, that OSM was able to upregulate TGF-β and PDGF in both resident and bone marrow-derived macrophages, suggesting an indirect pro-fibrogenic role in activating HSC/MFs to express collagen and TIMP-1 synthesis. This study is worth mentioning also because it provided additional evidence showing that the continuous expression of OSM in normal mouse liver, obtained by means of hydrodynamic tail vein injection, resulted in severe fibrosis [113], further confirming the pro-fibrogenic role of this cytokine.

More recently, we confirmed and extended the knowledge on the profibrogenic action of OSM. In particular, we showed that OSM and OSMβR were overexpressed in either liver specimens from NASH patients as well as in liver samples from three different dietary murine protocols effective in inducing experimental NASH, including methionine- and choline-deficient (MCD) diet, choline-deficient amino acid-refined (CDAA) diet and the high fat–high fructose (HFHF) diet [114]. Moreover, we provided evidence that OSM was able to directly stimulate the migration of human MFs through a biphasic mechanism that, similar to what shown for other chemoattractants acting on these cells [79,80], involved early generation of ROS, activation of the Ras/Erk, JNK1/2, PI3K/Akt, in addition to the STAT1/STAT3 pathways, and a late HIF-1α-dependent increased expression of VEGF-A [114]. We are currently investigating in more detail the pro-fibrogenic and pro-angiogenic action of OSM in relation to NAFLD progression and HCC development.

### 4.4. HIF-2α, HIF2 and CLD Progression: Relevant for Progressive NAFLD

As mentioned, the involvement of HIF-1α and then HIF1 in the progression of NAFLD and also of ALD is quite controversial, particularly in terms of the development of fatty liver, although the evidence for a pro-fibrogenic role is likely to be more robust, at least in relation to biliary fibrosis.

The role of HIF-2α and HIF2 has been deeply investigated only in recent years and most of the available data are indeed related to NAFLD fibrogenic progression, although HIF-2α was previously suggested as a critical mediator able to stimulate lipid storage [44,45,55]. In particular, one should emphasize that in the liver and in the scenario of a metabolic disease (NAFLD essentially develops in obese and/or type 2 diabetic patients), HIF-2α has a critical role in the control of glucose and fatty acid metabolism [123].

Indeed, the first mechanistic study supporting a role for HIF-2α in lipid metabolism employed hepatocyte-specific double knockout mice for VHL and, alternatively, HIF-1α or HIF-2α. This strategy revealed that only VHL/HIF-1α double knockout mice, resulting in a selective and constitutive activation only of HIF2α, developed severe hepatic steatosis and steatohepatitis in association with impairment of fatty acid oxidation, downregulation of lipogenic gene expression and increased lipid storage capacity [124]. These results were confirmed in a homologous study adopting a very close strategy of genetic manipulation and again constitutive HIF-2α (not HIF-1α) activation resulted in a rapidly developing severe steatosis. In addition, these authors reported also upregulation of genes involved in fatty acid synthesis/uptake and lipid storage and downregulation of those involved in fatty acid catabolism [125]. The latter study, in which mice were fed an ethanol-supplemented liquid diet for 2 weeks, also reported data indicating that constitutive activation of HIF-2α (i.e., VHL/HIF-1α double knockout mice) also led to increased transcription of pro-inflammatory cytokines (IL-1β and IL-6) and an initial condition of steatohepatitis. The same study also reported that constitutive activation of HIF-2α also resulted in increased transcription of the pro-angiogenic mediator angiopoietin-related protein 3 (ANGPTL3), an endogenous lipoprotein lipase inhibitor and an important mediator of lipid homeostasis. Moreover, HIF-2α overexpression also led to upregulation of some pro-fibrogenic genes, including lysyl oxidase-like 1 (LOXL1), lysyl oxidase-like 2 (LOXL2), prolyl 4-hydroxylase alpha 1 (P4HA1), prolyl 4-hydroxylase alpha 2 (P4HA2), procollagen-lysine, 2-oxoglutarate 5-dioxygenase 2 (PLOD2) as well as αSMA and collagen-1a1. A moderate increase in focal areas of fibrosis was also observed morphologically and this was of interest since usually the ethanol supplemented liquid diet administered to these mice does not elicit any detectable sign of fibrosis [125].

It should be noted, however, that these two elegant studies, although very indicative and mechanistic, were able to induce a steatosis, steatohepatitis or even initial fibrosis in conditions that are far from the human ones—animals carrying multiple genetical manipulations never occurring in NAFLD patients—and in the absence of significant liver injury [124], using the same approach associated with a very short protocol of ethanol administration [125].

In a subsequent study, it was for the first time described that HIF-2α was selectively overexpressed in the cytosol and the nuclei of hepatocytes in a very high percentage (>90%) of liver biopsies obtained from a cohort of NAFLD patients at different stages of the disease evolution. Moreover, the use of mice genetically manipulated to carry hepatocyte-specific deletion of HIF-2α fed a choline-deficient L-amino acid–defined (CDAA) or a methionine/choline-deficient (MCD) diet showed that these animals exhibited a very significant improvement in terms of a reduction in parenchymal injury, liver steatosis, inflammatory response as well as of fibrosis, as compared to wild-type littermates receiving the respective control diet [126]. The significant improvement in NAFLD progression detected in HIF-2α-deleted mice was related to a selective downregulation of the expression of histidine-rich glycoprotein (HRGP), a pro-inflammatory cytokine expressed by hepatocytes (i.e., a hepatokine) that was previously shown to be upregulated in both NAFLD and chronic hepatitis C patients and able to sustain M1 macrophage polarization and CLD progression [127]. Of interest, hepatocyte expression of HRGP was confirmed to be upregulated by hypoxia selectively through HIF-2α transcriptional activity. Even more relevant, analysis performed on human liver specimens from NAFLD patients showed the existence of a positive and significant correlation between HRGP and HIF-2α transcripts [126].

Increased liver expression of HIF-2α was confirmed in a more limited cohort of NASH patients in a subsequent paper and the same study also provided experimental evidence for a pro-inflammatory and profibrogenic role of HIF-2α, through involvement of NF-κB activation, in a model of “hypoxic” mice (i.e., mice housed at an altitude of 4300 m for several weeks and compared to the control animals housed at an altitude of 50 m) [128]. A critical role of HIF-2α upregulation was further confirmed in an additional experimental study employing mice fed on HFD and cells of a hepatocyte lineage manipulated in vitro. Once again lipid accumulation was found to be related to HIF-2α overexpression under hypoxic conditions and it was proposed that this could be related to the suppression of the PPARα target genes and mitochondrial impairment. In this study, there was a further confirmation that hypoxia, and selectively HIF-2α, was downregulating the expression of genes involved in fatty acid β-oxidation but upregulating those related to lipogenesis [129].

More recently, it has been reported that SerpinB3, a serine protease inhibitor produced by hepatocytes exposed to hypoxia selectively through HIF-2α transcriptional activity and found to be upregulated during CLD progression and HCC development [130,131], was able to exert a pro-fibrogenic action [132]. The profibrogenic action of SerpinB3 has been outlined using transgenic mice manipulated to overexpress SerpinB3 in hepatocytes and submitted to two distinct protocols for induction of liver fibrosis, namely, chronic CCl_4_ treatment and the NASH-related MCD diet. In both experimental models, SerpinB3 overexpression significantly worsened liver fibrosis and this was related to a net increase in the transcript levels of pro-fibrogenic genes, as well as of collagen deposition and the number of αSMA-positive HSC/MFs as compared to wild-type mice. Further in vitro experiments provided evidence that human recombinant SerpinB3 was able to strongly upregulate the expression of pro-fibrogenic genes and promoted oriented migration of either human HSC/MFs or human LX2 cells.

As a final comment, one should note that the mentioned involvement of HIF2α activation in pro-fibrogenic progression of CLD has already led to the use in preclinical studies of specifically designed therapeutic strategies. The first study to be mentioned employed in vivo administration of isoform-specific HIF-1α and HIF-2α antisense oligonucleotides (ASOs) in a murine model of diethyl-nitrosamine (DEN)-induced CLD and HCC [133]. This study revealed that both the ASOs employed did not prevent but rather enhanced inflammation and fibrosis, suggesting that directly targeting HIF2α in the liver may not represent the right approach. Indeed, if the pro-steatosis, pro-inflammatory and profibrogenic role of HIF2α in the liver has been unequivocally shown, it should be noted that HIF-2α activation can also significantly ameliorate hyperglycemia, either through the insulin-dependent pathway, with increased insulin receptor substrate-2 (IRS2), or the insulin-independent pathway, with the repression of glucagon action [134,135,136]. In other words, a therapeutic strategy designed to directly inhibit liver HIF-2α might not be suitable as a therapy for NAFLD, resulting in an increased risk to elevate hepatic glucose production and thus aggravate type 2 diabetes [42]. Along these lines, a very interesting study has provided consistent and mechanistic evidence that HIF2α involvement in obese and type 2 diabetes patients (i.e., the typical patients that develop progressive NAFLD) is much more complex [137]. In this study, the authors first showed mechanistically the relevance of intestinal HIF-2α activation for the development of experimental obesity, insulin resistance and hepatic lipid metabolism in a murine model [137]. Even more relevant, the authors also showed that the oral administration of PT2385, a selective HIF-2α antagonist that inhibits its transcriptional activity by blocking the heterodimerization between HIF-2α and HIF-1β, significantly prevented and reversed obesity and liver steatosis [137].

## 5. Therapeutic Strategies to Target HIFs or HIF-Related Processes and Pathways: Promising but Not Liver Oriented

**Figure 3:**
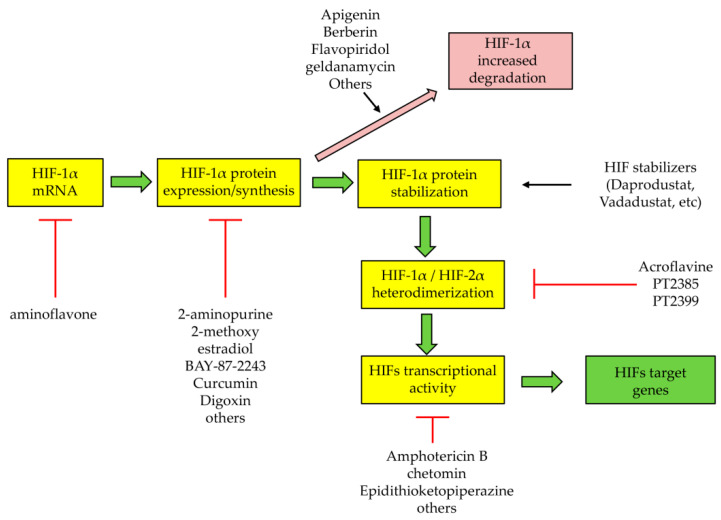
Site of action of drugs, small molecules and other agents acting either as HIFα subunit inhibitors or stabilizers. The site of action is shown in the ideal sequence of events, ranging from the HIFα subunit mRNA to the transcriptional activity of heterodimeric HIFs. Please note that the indicated drugs/small molecules/agents just represent a small selection of those developed in these years. For a more comprehensive list and detailed analysis, please refer to Reference [142].

The mentioning of the HIF-2α inhibitor PT2385 provides an occasion to introduce this section as dedicated to the established and emerging therapeutic strategies designed to specifically affect HIFs or HIF-related processes. PT2385 was originally developed and tested as a small molecule for the treatment of human clear cell renal cell carcinoma [138,139]. This small molecule, and likely its novel analog PT2399, is at present the only reliable example of a selective therapeutic approach that can directly and efficiently target HIF-2α, impeding its dimerization with HIF-1β and then the transcriptional activity of HIF-2. Indeed, one should consider that dozens of drugs have been tested in the past that, apart from PT2385, can indirectly affect HIFs and, as suggested by Semenza, can be generally divided into two categories: HIFs stabilizers and HIFs inhibitors [140]. Their mode of action is summarized in Figure 3.

HIF stabilizers are drugs that can pharmacologically stimulate HIF activity and are actually tested in clinical trials for the treatment of anemia due chronic kidney diseases (ACKD) and will be probably tested in the future, due to the positive results obtained in pre-clinical studies regarding their possible effect on intestinal bowel diseases [140]. Concerning anemia treatment, most of these drugs are small molecules acting as prolyl hydroxylase inhibitors. With their action they can stabilize the HIF-α subunits and increase HIF-dependent expression of erythropoietin. The first compound tested, active in a preclinical study, was dimethyl-oxalyl-glycine [141], but several other small molecules have been entered into clinical trials for ACKD, such as Daprodustat, Molidustat, Roxadustat, Vadadustat and others [140], with Daprodustat and Vadadustat recently approved for ACKD treatment in Japan [142,143]. Unfortunately, no data are available, to our knowledge, in relation to the use of these small molecules for the treatment of CLDs.

Concerning the HIF inhibitors, a very long list of small molecules and other drugs have been tested in order to target HIFs in relation to the established role of hypoxia and HIFs in carcinogenesis [140,144,145]. According to Semenza [140], these drugs can act by (see Figure 3) (i) decreasing HIF-1α transcription (aminoflavone) or protein expression (such as 2-amino-purine, 2-methoxy-estradiol, BAY-87-2243, curcumin, digoxin and several others); (ii) increasing HIF-1α degradation (such as apigenin, berberin, flavopiridol, geldanamycin, etc.); (iii) decreasing HIF-1α or HIF-2α heterodimerization (such as acriflavine, PT2385, PT2399); and (iv) decreasing HIF-1α transcriptional activity (amphotericin B, chetomin, epidithioketopiperazine, etc.) or DNA binding activity (doxorubicin, echimomycin and pyrrole-imidazole polyamide). These drugs have been tested in experimental models of carcinogenesis and in some clinical trials [40,99,140,146], but once again no one has been tested specifically to counteract fibrogenic progression of CLD. Moreover, one should also note that almost all of these drugs exhibit also other biological/pharmacological effects unrelated to HIFs.

In addition to these HIFs-stabilizers or inhibitors, it is worthy to recall that several laboratories have tried to employ anti-angiogenic therapies to counteract fibrogenic progression of CLD. This was a rational approach considering that pathological angiogenesis is an obvious hypoxia-dependent process and, as mentioned in a previous section, effectively develops in parallel with fibrogenesis (and, likely, can sustain it) in ongoing CLDs. To make the story short, several studies reported positive and sometimes very promising results in pre-clinical studies of antiangiogenic therapeutics. However, clinical trials that tested the most promising antiangiogenic drugs or antiangiogenic therapeutic strategies emerged from preclinical studies could not report any significant anti-fibrogenic efficacy in human CLD patients [29]. The antiangiogenic therapeutic strategies are now mostly limited to experimental carcinogenesis and clinical trials devoted to counteracting specific cancers [140].

## 6. Antifibrotic Therapies for CLD: A Synthetic Overview

In the last two decades, hundreds of preclinical studies have tested the efficacy of several putative antifibrotic drugs and therapeutic strategies, as detailed in a number of authoritative and exhaustive reviews on this topic [4,6,8,9,12,29]. Here, we would like simply to recall some of the most relevant principles and concepts that have emerged in these years and then some of the most promising approaches. However, we have to immediately state that, at present, as very recently emphasized by Scott Friedman and coworkers, there are no drugs specifically approved to treat hepatic fibrosis for any human CLD, whatever the etiology [147,148,149]. Before briefly analyzing the current therapeutic approaches emerging from preclinical studies, one should take in mind that, whenever possible, the most effective strategy to affect CLD progression remains to eliminate the etiological agent or condition leading to chronic liver injury. The most outstanding examples are represented by either the tremendous efficacy of direct antiviral agents (i.e., against HBV- and HCV-elated CLD) or by the long-term results obtained by abstinence from alcohol consumption. For all other CLDs, these options do not exists, and, as suggested by different authors, anti-fibrotic therapeutic strategies and therapeutic targets may be briefly analyzed according to the following critical options, as recently outlined particularly for progressive NAFLD [4,5,6,8,9,147,148,149,150].

### 6.1. Strategies to Reduce Liver Parenchymal Injury

This approach, proposed by extensive pre-clinical data obtained in the last two decades, includes the use of the following agents able to reduce hepatocyte injury [4,5,6,150,151,152]: (i) antioxidant molecules, such as vitamin E, *N*-acetyl-cysteine, resveratrol, curcumin and herbal supplements, designed to protect from oxidative stress-related cell injury and death; (ii) NADPH-oxidase inhibitors, designed to prevent intracellular generation of ROS and then oxidative stress; and (iii) the use of pan-caspase inhibitors, such as emricasan and PF-03491390. Emricasan is at present in a Phase II trial for NASH patients (NCT02686762). Waiting for the emricasan effects in humans, these agents were generally very effective in experimental models of CLDs but offered very modest and transient improvements, if any, when tested in clinical trials [4,5,6,150,151,152,153].

### 6.2. Strategies to Target Hepatic Macrophages

Another promising approach emerged in recent years is represented by strategies targeting liver macrophages (either Kupffer cells or monocyte/macrophage from peripheral blood) as well as those signaling pathways underlying their recruitment and/or activation [12,13,149,153]. Pre-clinical studies have identified a number of critical targets for this anti-inflammatory purpose, including the CCR2/5 pathway, the serine/threonine apoptosis-stimulated kinase 1 (ASK1), several Toll-like receptors (TLR), galectin 3, Adenosine A3 receptor, vascular adhesion protein 1 (VAP-1) and others. Worthy of mention are at least four drugs: (i) Cenicriviroc, a double inhibitor of the CCR2/5 pathway that alone (NCT03028740) has entered into Phase III trials for NAFLD patients or it is under evaluation in combination with tropifexor, a potent farnesoid X receptor (FXR) agonist [149]; this drug is of potential interest since it can also target HSC/MFs and not only liver macrophages. (ii) Selonsertib, a selective inhibitor of ASK1, expected to block inflammation and fibrosis through necrosis and apoptosis, which was found to be ineffective in NASH patients with Stage 3 fibrosis and cirrhosis in two distinct Phase III trials (NCT03053063 and NCT03053050) [149]. (iii) The VAP-1 inhibitor BI 1467335, which is at present studied in a Phase II trial (NCT03166735) and expected to reduce inflammation and then fibrosis. (iv) JKB-121, which is an antagonist of TLR4, a receptor known to be relevant for the activation of inflammatory cytokine signaling and liver injury; this drug is now tested in a Phase II trial (NCT02442687) enrolling patients with biopsy-proven NASH and is expected to inhibit the TLR4-dependent inflammatory cascade as well as HSC activation, and the formation of collagen (TLR4 is expressed also by hepatic MFs) [149].

### 6.3. Strategies to Target MFs and to Inhibit Fibrosis through Different Approaches

Although the strategy to target MFs may seem quite obvious, it should be noted that most of the pre-clinical studies (few translated into clinical trials) have been directed to modulate the activating signals or intracellular cascades involved in eliciting or perpetuating MF activation. Several agents/molecules have been tested and here it is worth mentioning just some of these in addition to those already mentioned before (i.e., cenicriviroc and JKB-121) [4,5,6,8,9,147,148,149]. Major targets are here represented, for example, the nuclear receptor signaling pathways, such as FXR, peroxisome proliferator-activated receptor (PPAR)-α, PPAR-γ and PPAR-δ, vitamin D receptor, liver X receptor and others. Agonists of these receptor have been shown to inhibit HSC activation to the MF-like phenotype and, in experimental models, to prevent or reduce fibrosis. Some of these agonists, particularly those for PPARs and FXR, have been tested in clinical trials also because of their proposed ability to interfere with mechanisms leading to steatosis. Concerning PPAR agonists, there are three different molecules in Phase II trials in NAFLD/NASH patients, such as Saroglitazar, which is a PPAR α/γ agonist (NCT03061721), Lanifibranor, a PPARα/γ/δ agonist (NCT03008070), and Seladelpar, a PPAR δ agonist (NCT03551522). Another PPAR α/δ agonist, Elafibranor, showed no effect in a Phase III clinical trial still involving NASH patients. FXR agonists, such as obeticholic acid and EDP-305, have entered into Phase III (NCT02548351, NCT03439254) and Phase II (NCT03421431) clinical trials, respectively, in NASH patients, although the best results for obeticholic acid have been reported for PBC patients.

Another strategy designed to target MFs has been to interfere with those dysregulated pathways contributing to MF persistent activation. Typical examples are the attempt to interfere with the signaling pathways elicited by TGFβ1, PDGF, CTGF or other signaling pathways involving ligand/receptor interaction, which are all able to sustain the MF phenotype. At present, none of these approaches have been translated into clinical trials. Finally, one has to briefly mention agents that recently entered into clinical trials for NASH patients [149], including the following: (i) Resmetirom/MGL-3196, a selective agonist of THR-β (thyroid hormone receptor, currently in a Phase III trial (NCT03900429), expected to attenuate the severity of NASH; and (ii) FGF-21 analogues, expected to improve glucose homeostasis and insulin sensitivity as well as exert anti-fibrotic potential; it should be mentioned here two of these compounds, such as agent BIO89-100 and efruxifermin, are both currently tested in Phase II clinical trials (NCT04048135 and NCT03976401, respectively).

## 7. Conclusions

In the present review we have offered an update on the studies concerning the potential role of hypoxia and HIFs as well as of major HIF-related mediators in the fibrogenic progression of CLD. In physiological conditions the liver has a unique double vascular supply by receiving oxygen-depleted blood from portal vein branches and highly oxygenated blood via the hepatic artery, subsequently generating a gradient of oxygen partial pressure between the periportal and perivenous areas. However, even modest changes to the liver architecture, as in progressive CLDs, due to persisting/sustained parenchymal injury, inflammatory response and excess deposition of ECM components and vascular reorganization, can significantly result in tissue hypoxia and a hypoxic cellular response. Emerging recent evidence is revealing that the two major HIFα subunits, HIF-1α and HIF-2α (and then HIF-1 and HIF2), can both respond to hypoxia but their transcriptional gene targets, oxygen dependence and kinetics of activation can differ substantially. Data emerging from preclinical and clinical studies are starting to delineate a scenario in which HIF-1α and HIF-2α may operate differently in relation to CLD progression, with a recent focus mainly on progressive NAFLD and ALD. This bulk of knowledge is now flanked by the emergence of novel and more selective drugs that may target either HIFs, a component of the related transcription machinery or directly the HIF-controlled genes, an issue that may open the way to design novel therapeutic strategies to inhibit CLD progression. This is an absolute requirement since, although several promising anti-fibrotic agents are being evaluated in Phase II and Phase III trials, at present there are no drugs specifically approved to treat hepatic fibrosis in human patients.

## Figures and Tables

**Table 1:** Chronic liver diseases in human patients. Major etiological agents/conditions, type of disease and patterns of fibrosis.

Type of Chronic Injury	Agent/Condition	Disease	Pattern of Fibrosis
Parenchymal: viral infection	HBV		
	HCV	CVH	Bridging fibrosis, post-necrotic
	HBV + HDV		
Parenchymal: altered metabolism	Obesity/T2DM/MS	NAFLD	Pericellular/perisinusoidal fibrosis
Parenchymal: toxic	Ethanol	ALD	Pericellular/perisinusoidal fibrosis
Parenchymal: immune-mediated	Autoimmune injury to hepatocytes	AH1	Bridging fibrosis,
		AH2	post-necrotic
Parenchymal: genetically related		α1AT	
	Hereditary	WD HH	Bridging fibrosis, post-necrotic
Cholangiopathies: genetically related		Alagille syndrome	
	Hereditary	Caroli syndrome ABCB4 deficiency Cystic fibrosis Polycystic disease	Biliary-like fibrosis
Cholangiopathies: immune-mediated	Autoimmune injury to cholangiocytes	PBC PSC	Biliary-like fibrosis
Cholangiopathies: unknown	Idiopathic cholangiopathies	Biliary atresia Sarcoidosis	Biliary-like fibrosis

Abbreviations: ALD: alcoholic liver disease; α1AT: α1-anti-trypsin disease; AH1: autoimmune hepatitis 1; AH2: autoimmune hepatitis 2; HBC: hepatitis B virus; HCV: hepatitis C virus; HH: hereditary hemochromatosis; NAFLD: non-alcoholic fatty liver disease; PBC: primary biliary cholangitis; PSC: primary sclerosing cholangitis; WD: Wilson′s Disease.

## Abbreviations

ACKD: anemia of chronic kidney disease; ALD: alcoholic liver disease; ALT: alanine aminotransferase; ANGPTL3: angiopoietin-related protein 3; ASK1: apoptosis-stimulated kinase 1; αSMA: α-smooth muscle actin; ARNT: aryl hydrocarbon receptor nuclear translocator; bHLH-PAS: basic helix–loop–helix Per–Arnt–Sim; ASOs: antisense oligonucleotides; BNIP3: BCL2/adenovirus E1B 19 kDa protein-interacting protein 3; BDL: bile-duct ligation; BNIP3L: BNIP3 ligand; CBP: cAMP-response element binding (CREB)- binding protein; CDAA: choline-deficient amino acid-refined; CLD: chronic liver disease; CTGF: connective tissue growth factor; DEN: diethyl-nitrosamine; DLL4: delta-like ligand 4; ECM: extracellular matrix; EMT: epithelial-mesenchymal transition; ER: endoplasmic reticulum; EVs: extracellular vesicles; FGF: fibroblast growth factor; FIH1: factor inhibiting HIF1; FXR: farnesoid X receptor; GLUT1: glucose transporter 1; HBV: hepatitis B virus; HCC: hepatocellular carcinoma; HCV: hepatitis C virus; HFHF: high fat–high fructose; HIF: hypoxia-inducible factor; HRE: hypoxia responsive elements; HRGP: histidine-rich glycoprotein; HSC: hepatic stellate cells; HSC/MFs: activated, MF-like hepatic stellate cells; iNOS: inducible NO synthase; IRS2: insulin receptor substrate 2; LCAD: long-chain acyl-CoA dehydrogenase; LDHA: lactate dehydrogenase A; LIFRβ: leukemia inhibitor factor receptor β; LZIP: leucine zipper; LOXL: lysyl oxidase-like; MAPK: mitogen activated protein kinase; MCAD: medium-chain acyl-CoA dehydrogenase; MCD: methionine and choline deficient; MFs: myofibroblasts; MMP13: matrix metalloprotease 13; mTOR: mammalian target of rapamycin; NAFLD; non-alcoholic fatty liver disease; NASH: non-alcoholic steatohepatitis; NDRG-2: N-Myc downstream-regulated gene 2; NF-κB: nuclear factor kB; NK: natural killer; NDUFA4L2: NADH dehydrogenase [ubiquinone] 1α subcomplex, 4-like 2; NOX: NADPH oxidase; OSM: oncostatin M; OSMRβ: OSM receptor β; PBC: primary biliary cholangitis; PDGF: platelet-derived growth factor; PDK1: pyruvate dehydrogenase kinase 1; PGC-1β: peroxisome proliferator-activated receptor-gamma (PPAR-gamma) coactivator-1 beta; P4HA: prolyl 4-hydroxylase alpha; PLOD2: procollagen-lysine, 2-oxoglutarate 5-dioxygenase 2; PPAR: peroxisome proliferator-activated receptor; PHD: prolyl hydroxylase; PSC: primary sclerosing cholangitis; REDD1: regulated in development and DNA damage responses 1; ROCK1: Rho associated protein kinase 1; ROS: reactive oxygen species; SEC: sinusoidal endothelial cells; SOCS: suppressor of cytokine signaling; SOD2: superoxide dismutase 2; STAT: signal transducer and activator of transcription; TGFα: transforming growth factor-α; TGFβ: transforming growth factor β; TIMP1: tissue inhibitor of metalloproteinase 1; VAP-1: vascular adhesion protein 1; VEGF: vascular endothelial growth factor; VEGFR2: VEGF receptor 2; VHL: von Hippel–Lindau.
